# What Do General Practitioners Want?

**Published:** 1897-10-02

**Authors:** 


### The Hospital

A Journal of

The Medical Sciences and Hospital Administration.

Vol. XXIII.—No. 575.] [Oct. 2, 1897.

### What Do General Practitioners Want?

In view of the coming election of a direct representative on the General Medical Council, it is incumbent on the general practitioners of medicine to make up their minds definitely and quickly as to what it is they want.

The first question which they have to decide is whether they are mostly attached to men or to measures. No doubt there is a strong feeling abroad that, the corporations being almost exclusively represented by men of consultant rank, the direct representatives should in all equity be chosen from among those who always must form the great majority of the constituency, namely, the general practitioners. It is a reasonable desire, and one against which we have not a word to say, but the question is, Is that the main desire which is felt by the general practitioners of the country? From their numbers they have the ball absolutely at their feet if they can but make up their minds and be unanimous, and we ask, Is this what they want?—to be represented in the Council by a fellow general practitioner?—or do they not rather desire to enforce certain measures and to advance certain reforms? If the latter is what they desire then they should select the man who is most likely to help them to get it, quite irrespective of his position, his place of residence, or his department of practice.

What is plain to outsiders, however little apparent it may be to those who are within the charmed circle, is that the Council might be made a very much more effectual instrument for advancing the interests of the profession than it is at present; but to effect this change in its functions, this development of its powers, what is required is something very different from a mere increase in the number of members likely to vote in favour of reform. Some of the most respectable nonentities in Parliament are the best voters, and thus the most useful of party men; and if the drift of affairs of the Medical Council were to be decided by mere votes, a steady-going man, with a definite mandate, would, no doubt, be a sufficiently competent representative of the medical profession. We have to recognise, however, that so far as voting power is concerned the interests of the body of the profession are, and will for long to come, be represented on the Council in so insignificant a manner that even if the direct representatives were always to vote solid on every question it would only be on the rarest occasions that they would be able to exercise any effective influence on the proceedings. What we want is a man of initiative and of proved business capacity, and one able to compel attention to the wants of his constituents, rather than a mere voter.

Practically there are but two men before the profession, viz., Mr. Victor Horsley and Sir Walter Foster. Both of them are good men and, so far as their addresses go, there seems but little to choose between them in regard to the measures which they are prepared to support. What Sir Walter Foster has done for the profession, more especially in Parliament, must have weight with us all. The very fact that he is in Parliament, and is thus able to voice the wants of the profession in the House of Commons, is also much in his favour, and in addition to all this we believe that he would be a *persona grata* to the majority of those now having seats on the Council.

Sir Walter Foster has, however, been through the official mill; he has been much in contact with that admirable but depressing person the British permanent official, and has had his asperities rubbed off, and the profession has to consider whether these are really advantages to a man who would fully represent and consistently enforce the wants of the medical profession on a reluctant council.

Mr. Victor Horsley's claims stand on quite a different basis. So far as the *personnel* of the Council goes, he is entirely unfettered, while his knowledge of the wants of the profession and his proved willingness to further its interests, qualify him in a peculiar manner for becoming its direct representative. His platform is not wide, but it is perfectly definite, and it embraces the very points which general practitioners feel are of the most vital importance for their welfare. Of these, perhaps, the most urgent is that the powers which the Council undoubtedly possesses for the punishment of those who transgress professional rules, in regard especially to medical aid societies, should be put in force; the next is that the Medical Acts should be amended in regard to (*a*) the representative constitution of the Council, (*b*) the securing of a one-portal system of admission to the profession, and(*c*) the suppression of irregular practice by confining practice for gain to registered persons.

These, we take it, are the points in which the practitioners of to-day take the greatest interest, and on which they are practically unanimous; and for the carrying of these points Mr. Victor Horsley is by far the most powerful candidate in the field. Of all agencies for stirring up the placid serenity of the General Medical Council, the Medical Defence Union has proved much the most effective, and it would seem peculiarly appropriate that the President of that union should be made the mouthpiece of the profession in the deliberations of that august body.

